# The Story of the Sun, Moon, and Stars

**Published:** 1900-11

**Authors:** 


THE STORY OF THE SUN, MOON, AND STARS. By Agnes Giberne, author of "Radiant Suns," "The Starry Skies," "The World's Foundatoins," etc. With an introduction by Charles Pritchard, M. A., F. R. S., Professor of Astronomy in the University of Oxford. With copious additions.

We have derived much pleasure and satisfaction from reading this great book, for it *is* a great book. Miss Giberne has done a real benefaction in telling of the scientific revelations concerning the universe in a style divested of technical terms, yet so pleasing as to invest the subject with the charm of romance. It is adapted to the simplest understanding, and yet holds the attention even of scholars who are familiar with the history and discoveries of astronomy. We can and do commend the work highly, and think it would be well if it were introduced into the schools, not as a text-book on astronomy, but as a "reader." All young persons, especially, should read it. It is intended for those who wish to hear an account of the things which surround our earth, and would like to acquire, without hard work, an elementary and exact idea of the present condition of the universe. It is a picturesque delineation of the heavens; a work of vast research and scholarship; but these qualities are never obtruded upon the attention of the reader. They are hidden away under a smooth and flowing style, that carries with it an unflagging interest. Such is the clearness of the narrative, such its brilliancy and elevation, that one forgets, as he peruses these pages, the years of patient labor devoted to the preparation of the work. The author's knowledge of the subject is exact, her style is admirable for its grace, clearness and vivacity, and possesses like a sparkling wine, qualities peculiar to itself.

In one handsome volume of some 450 pages, clear type, on good paper, well and substantially bound in cloth, and the price is $1.50. Published by the National Book Company, 437-441 Race Street, Cincinnati, Ohio. Copiously and well illustrated.

